# Efficacy of high-volume vs very low volume corticosteroid subacromial injection in subacromial impingement syndrome: a randomized controlled trial

**DOI:** 10.1038/s41598-023-29449-4

**Published:** 2023-02-07

**Authors:** Adinun Apivatgaroon, Surasak Srimongkolpitak, Phanuwat Boonsun, Bancha Chernchujit, Prakasit Sanguanjit

**Affiliations:** Department of Orthopaedics, Faculty of Medicine, Thammasat University, Bangkok, Thailand

**Keywords:** Diseases, Medical research, Signs and symptoms

## Abstract

Subacromial corticosteroid injections (SCI) treat shoulder pain from subacromial impingement syndrome (SIS). However, a high-volume corticosteroid injection (HVCI) increases the incidence of local and general complications from lidocaine. This study aimed to compare the results of functional and clinical outcomes between the very low-volume corticosteroid injection (VLVCI) and HVCI including to WORC (Western Ontario Rotator Cuff Index), VAS (Visual Analog Scale), DASH (The disabilities of the arm, shoulder, and hand) and ROM (Range of Motion). A total of 64 patients presenting with SIS were evaluated in two SCI volume in a randomization-controlled trial study. The VAS for pain pre-injection and post-injection at 15 min, was from 5.34 ± 2.44 at before injection to 2.44 ± 1.58 at post injection 15 min in the HVCI group (P < 0.001) and from 5.19 ± 2.33 to 2.84 ± 1.49 in VLVCI group (P < 0.001). Not significant differences at mean difference VAS post-injection 15 min VAS (P = 0.324) and Percentage difference VAS pre-injection and post-injection (P = 0.24). All follow-up timing, there were no significant differences in WORC, DASH and ROM between two groups (P > 0.05). The results revealed the VLVCI is non-inferior to HVCI both of functional outcomes and VAS.

## Introduction

Subacromial impingement syndrome is one of the most common shoulder diseases, and it can lead to significant limitations in activities and lifestyle modifications^1^. Subacromial impingement syndrome (SIS) is one of the most common diagnoses for shoulder disorders causing subacute to chronic shoulder pain. Its prevalence ranges from 36 to 74% of patients from the shoulder pain syndrome, with an average of approximately 7% to 34%^2,3^. The first line of treatment for SIS is oral medicines, physical therapy, or subacromial injections which an oral medication and physiotherapy still utilized in management of impingement syndrome. Subacromial injections have a disease-modifying effect by reducing inflammation and enabling physical therapy, in addition to relieving pain and improving patient comfort^4^. When compared to oral medication or rehabilitation, the SCI is superior in terms of short duration, improved pain score, and ability to perform both therapeutic and diagnostic processes because it directly decreases inflammation at the subacromial space.

However, there is a lot of variability in the corticosteroid injection technique, and one explanation of variability could be the method by which corticosteroid injections are administered, such as being given aim various doses (low or high), volume (low or high), and the injection technique^5^. These issues that are inconclusive. There has been no research that compares the varied volumes of corticosteroid injection in earlier published studies, and there is no alternative group of intervention that can be utilized to suggest that lidocaine has no effect in treatment SIS, such as pure corticosteroid injection^6^.

The patient still found the main complication of lidocaine combined with corticosteroids to be more neurotoxicity after subacromial injection with the standard SCI mixed solution with anesthetic solution (lidocaine) and corticosteroid solution. The main complication of the patient was where lidocaine was combined with corticosteroids resulting in more neurotoxicity after subacromial injection. Slurred speech, tinnitus, circumoral paresthesia, feeling faint (vasovagal syncope), seizures or loss of consciousness, cardiac arrhythmias, respiratory arrest, and cardiac arrest, all seem to be signs and symptoms of lidocaine toxicity^7^. Even if the resolution of these problems is observation, and continuous monitoring, all complications continue to cause problems and affect the patient degree of satisfaction.

Generally, when compared to low-volume CI, HVCI of lidocaine is associated with a higher risk of consequences, in particular, soft tissue irritation. And furthermore, when corticosteroids are at lower concentrations, the steroid effects decrease, which should only be acceptable if being using for therapeutic purposed^8^. In the case of patient, the pain and functional outcomes of the corticosteroid injection were satisfactory. When compared to low-volume, HVCI had a reduced ASES, VAS, and risk of complication. However, no statistically significant differences occur between different groups^9^.

We hypothesized that VLVCI was not of significance to the functional score and VAS compared to in the HVCI. This study difference from the others studies due to aims to use VLVCI to increase the most potentially beneficial steroid concentration effect while avoiding the complications associated with lidocaine and compare the efficacy of corticosteroid injections in the SIS using HVCI and VLVCI (pure corticosteroid injection without analgesic solution combination).

## Materials and methods

The prospective randomized control trial was designed specifically for this study. Sixty-four patients were selected during August 2020–May 2021 from the Outpatient Clinic, Department of Orthopedics, Thammasat University Hospital. After the informed consent was obtained for each of the patients on the basics of the inclusion criteria, the data and follow-up of the patients were recorded. The procedure of subacromial corticosteroid injection for each patient was block randomized by the computer randomization program (www.sealedenvelope.com). All patients were injected by the fellowship training sport medicine orthopedic surgeon; the visual analogue scales before injection and after 15 min injection being recorded. The inclusion criteria comprised of the following: Impingement Syndrome from physical examination; x-ray and MRI; Impingement syndrome had failed conservative treatment for 6 weeks; age 20–70 years, absence of any calcific tendinitis as confirmed by x-ray or MRI findings; a negative Jobe’s test, and BMI < 30. The exclusion criteria comprised the following: adhesive capsulitis (forward elevation < 120 degrees); post trauma event; corticosteroid injection > 6 months earlier, and with external rotation < 60 degrees, glenohumeral joint arthritis, anticoagulant or antiplatelet affected to injection.

### Compliance with ethical standards

All procedures performed in the study involving human participants were done in accordance with, an adherence to the ethical standards of The Human Research Ethics Committee of Thammasat University Medicine and the study was approved by the ethics committee/IRB of MTU-EC-OT-0-182/63 on 03/12/2020. The Clinical Trial Registration: Thailand Clinical Trials Registry approved by TCTR Committee on 24/01/2021. The TCTR identification number is TCTR20210124001.

### Sample size calculation

The required sample size was determined by means of power analysis on the basis of a previous study of patients with “Short term outcomes of subacromial injection of combined corticosteroid with low volume compared to high volume local anesthetic for rotator cuff impingement syndrome: a randomized controlled non inferiority trial”^9^.

A power analysis program was used to calculate the number of participants required by; approximately 56 participants being required for repeated measures, with α of 0.05. A total of 64 participants was required to allow for 10% loss to follow-up. At the final analysis was analyzed power (1−β) of 80%.

### Data collection and evaluation

Sixty-four patients were enrolled and randomized with 32 patients being injected using the high-volume corticosteroid injection and the very low corticosteroid injection was used on 32 patients by a randomization block of 6. The patients and surgeons were inherently not blinded to the trial treatments. To ensure blinding of the outcome assessors. WORC, DASH, ROM, and complication at 2 weeks and 1, 3, 6 months follow-up were recorded only by blind assessors using sealed opaque envelopes. Assessors were an orthopedics fellow and staff at the Outpatient Clinic, Department of Orthopedics.

### Procedure protocol

**Figure 1:**
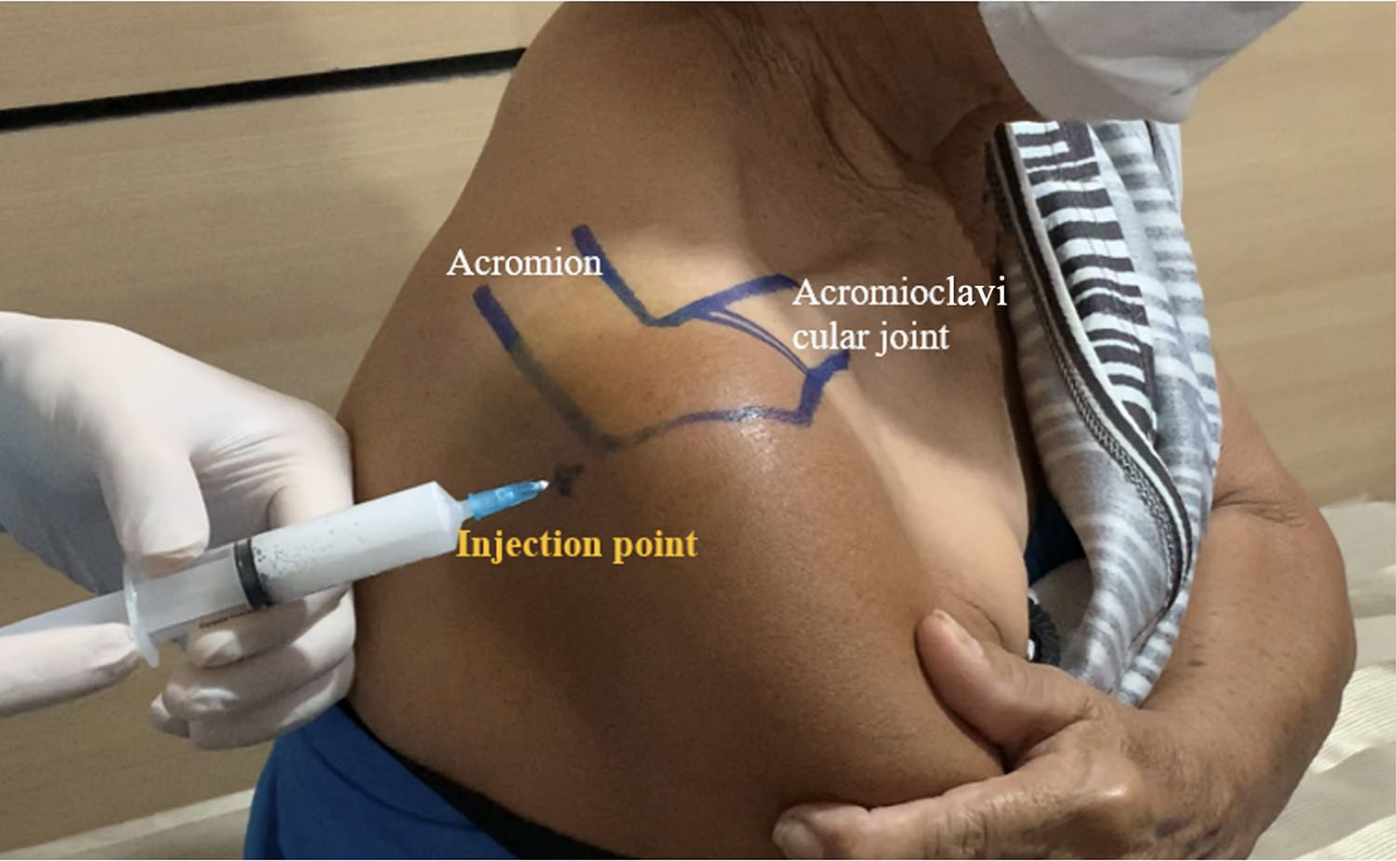
Landmark of the posterior approach of subacromial corticosteroid injection with demonstrated a posterior injection point.

The subacromial corticosteroid injection was performed using a posterior approach without ultrasound guidance. The affected shoulder was prepared with a Betadine solution. In the case of high-volume corticosteroid injection, a 10 ml syringe was filled with 40 mg triamcinolone acetonide 1 ml and 1% lidocaine hydrochloride without epinephrine 9 ml, and a very-low volume corticosteroid injection, a 1 ml insulin syringe was filled with 40 mg triamcinolone acetonide 1 ml without 1% lidocaine hydrochloride. A 23-gauge needle was used for injection. The bony prominence of the posterolateral corner acromion was palpated. The injection point was 2 cm, both inferiorly, and medially to the posterolateral corner acromion by posterior approach. The needle was introduced directly into the subacromial space, and angled at about 45 degrees, following acromion tilted (Fig. 1). The patient was allowed to take paracetamol for pain relief but was not any prescribed NSAIDs drug or opioid drug. The high-volume corticosteroid injection participants had to stay at the OPD for more than 20 min following the injection, allow time to detect any signs of acute adverse reactions that might occur, including local bleeding, weakness, anaphylaxis, and vasovagal reaction.

The primary outcome was WORC at pre-injection, and 1, 3 and 6 months. The patient was informed of the WORC information recorded. The follow-up period secondary outcomes recorded included: VAS at pre-injection, 15 min post-injection, 2 weeks, and at 1, 3 and 6 months; DASH at 1, 3 and at 6 months, and ROM with forward elevation and external rotation of the arm at the side, at pre-injection, at 2 weeks and 1, 3, and at 6 months.

### Statistical analysis

An unpaired t-test for continuous variables (age, grade) and chi-square was calculated by categorical variable (gender). The WORC, DASH and VAS were compared between two groups following a subacromial corticosteroid injection, by using unpaired t-test. 5. The repeated measurement ANOVA analysis is used to a longitudinal during follow-up in this study. Statistical significance was defined as being where a P-value was less than 0.05. The data analyses were performed using SPSS version 20.

## Results

**Figure 2:**
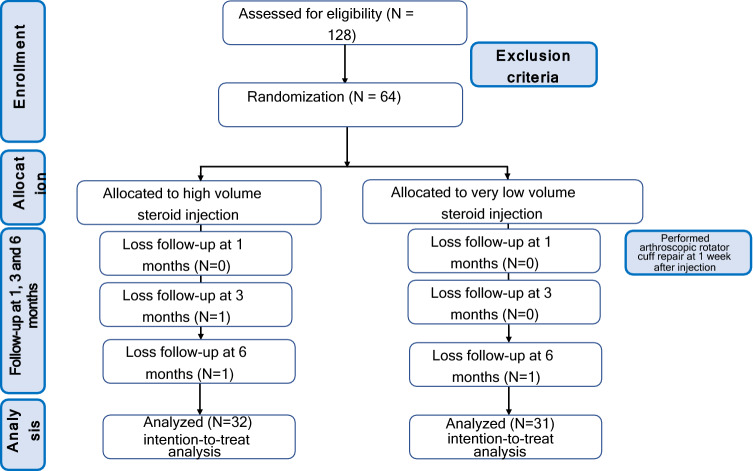
Consolidated Standards of Reporting Trial (CONSORT) diagram for the recruitment and flow of participants through the trial.

One hundred and twenty-three eligible patients were included in our study. Sixty-four patients (64 shoulders) were randomized, 32 patients (32 shoulders) for the HVCI, and 32 patients (32 shoulders) for the VLVCI (Fig. 2). 64 patients with subacromial impingement syndrome, (32 shoulders) for each volume corticosteroid injection, were included in the study. The Independent T-test or Mann–Whitney Test, and Chi-square test was used to analyses all the demographic data, including to age, sex, dominant side, affected side, duration, VAS, WORC and DASH. There were no statistical differences among the groups in relation to age, sex, dominant side, affected side, duration, VAS, WORC and DASH. The demographic and clinical data of patients for each group is shown in (Table 1).

**Table 1:** Demographic data detail of age, sex, dominant side, affected side, duration, VAS, WORC and DASH by treatment group.

		High volume (n = 32)	Very low volume (n = 31)	*P* value
Age means (SD)		57.72 ± 9.49	60.35 ± 7.38	0.224
Sex (%)	Male	11 (34.4%)	6 (19.4%)	0.179
	Female	21 (65.6%)	25 (80.6%)	
BW		64.78 ± 9.88	64.4 ± 8.98	0.874
BH		161.74 ± 5.99	160.97 ± 4.4	0.568
BMI		24.81 ± 3.6	24.87 ± 3.36	0.951
Affected side(%)	Right	14 (43.8%)	15 (48.4%)	0.712
	Left	18 (56.3%)	16 (51.6%)	
Dominant hand (%)	Right	29 (90.6%)	30 (96.8%)	0.317
	Left	3 (9.4%)	1 (3.2%)	
Duration (month) median (range)		3 (1–12)	3 (1–8)	0.057
VAS pre-injection, mean (SD)		5.34 ± 2.44	5.19 ± 2.33	0.804
WORC pre-injection, mean (SD)		43.6 ± 15.02	48.39 ± 15.71	0.221
DASH pre-injection, mean (SD)		49.53 ± 21.18	48.59 ± 16.83	0.845

### Primary outcomes

#### WORC score

**Figure 3:**
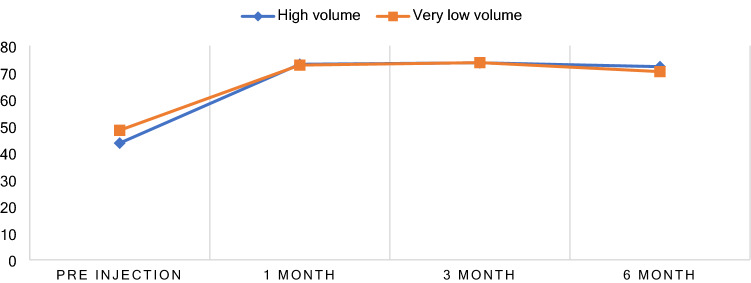
Mean WORC score by the progression time to treatment with SCI.

There was improvement of the WORC score in the case of each group 1, 3 and 6 months. The P-value analysis in WORC was determined by applying the Generalized Estimating Equation (GEE) in order to assesses the difference between the groups at any point in time; the significance level being set at 0.05. It was determined that there were no significant differences in WORC score between the 2 trial groups at any point in time. At pre-injection time, a WORC score of 43.6 ± 15.02 was recorded for the HVCI group. In the case of the VLVCI group a score of 48.39 ± 15.71 (P = 0.221) was recorded (Table 2). At 1 month follow-up, a WORC score of 73.05 ± 17.93 was recorded for the HVCI group. The VLVCI group, the WORC score recorded was 72.73 ± 11.63 (P = 0.934). After 3 months of the SCI, both groups reported a mean WORC score of 73.59 ± 13.68 for the HVCI, and 73.66 ± 11.89 in VLVCI, by patient. 3 months post-injection, the 3-month WORC score showed there to be no statistical difference in increased WORC score between of two groups (P = 0.984). Finally, on the 6-month follow-up on the SCI, the HVCI group and the VLVCI group each reported the WORC score as have increased to 72.15 ± 18.5 in the HCI group, and 70.3 ± 21.12 in VLVCI group, the implication being that there were no statistically significant differences in the WORC score (P = 0.712) (Table 3). An interesting finding was that the VLVCI group improved both in the midterm and the long-term WORC score, with there being no difference for the HVCI group or similar trend of progression outcomes (Fig. 3).

**Table 2:** Result of Mean of VAS, WORC and DASH within group at difference time compared after baseline, 2 weeks and 1-, 3- and 6-months follow-up.

		High volume group (n = 32)			Very low volume group (n = 31)		
		Mean ± SD	Within group Mean difference (95%CI)	*P* value	Mean ± SD	Within group Mean difference (95%CI)	*P* value
VAS	Pre-injection	5.34 ± 2.44	Reference	1	5.19 ± 2.33	Reference	1
	15 min post injection	2.44 ± 1.58	− 2.91 (− 3.76, − 2.05)	< 0.001*	2.84 ± 1.49	− 2.35 (− 3.09, − 1.62)	< 0.001*
	2 weeks	2.59 ± 1.79	− 2.75 (− 3.61, − 1.89)	< 0.001*	2.58 ± 1.18	− 2.61 (− 3.35, − 1.87)	< 0.001*
	1 month	2.47 ± 1.46	− 2.88 (− 3.73, − 2.02)	< 0.001*	2.61 ± 1.43	− 2.58 (− 3.32, − 1.84)	< 0.001*
	3 months	2.81 ± 1.26	− 2.53 (− 3.39, − 1.67)	< 0.001*	3.06 ± 1.61	− 2.13 (− 2.87, − 1.39)	< 0.001*
	6 months	3.56 ± 2.46	− 1.78 (− 2.64, − 0.92)	0.001*	3.03 ± 1.62	− 2.16 (− 2.9, − 1.42)	< 0.001*
WORC	Pre-injection	43.6 ± 15.02	Reference	1	48.39 ± 15.71	Reference	1
	1 month	73.05 ± 17.93	29.45 (21.89, 37.01)	< 0.001*	72.73 ± 11.63	24.35 (17.8, 30.89)	< 0.001*
	3 months	73.59 ± 13.68	29.99 (22.43, 37.55)	< 0.001*	73.66 ± 11.89	25.27 (18.72, 31.82)	< 0.001*
	6 months	72.15 ± 18.5	28.55 (20.99, 36.11)	< 0.001*	70.3 ± 21.12	21.91 (15.37, 28.46)	< 0.001*
DASH	Pre-injection	52.06 ± 17.76	Reference	1	48.59 ± 16.83	Reference	1
	1 month	26.02 ± 16.23	− 26.04 (− 33.93, − 18.16)	< 0.001*	23.68 ± 9.88	− 24.91 (− 31.49, − 18.32)	< 0.001*
	3 months	27.1 ± 15.52	− 24.96 (− 32.85, − 17.08)	< 0.001*	27.94 ± 13.76	− 20.65 (− 27.23, − 14.06)	< 0.001*
	6 months	24.4 ± 16.19	− 27.66 (− 35.55, − 19.78)	< 0.001*	28.11 ± 19.29	− 20.48 (− 27.06, − 13.89)	< 0.001*

* The data was statistically significant (*P*-value <0.005).

**Table 3:** Result of VAS during activity outcomes at pre injection, post injection 15 min, Percentage difference improvement after 15 min, 2 weeks and 1, 3 and 6 months, WORC at 1 month and DASH at 1, 3 and 6 months between high volume and very low volume corticosteroid injection.

Outcome at the follow-up	High volume (n = 32)	Very low volume (n = 31)	Mean difference between group	*P* value
			95% CI	
VAS pre-injection during activity	5.34 ± 2.44	5.19 ± 2.33	0.15 (− 1.05, 1.35)	0.804
Mean VAS difference between pre-injection and post-injection 15 min	− 2.91 ± 2.44	− 2.35 ± 1.92	− 0.55 (− 1.66, 0.56)	0.324
VAS post-injection 15 min	2.44 ± 1.58	2.84 ± 1.49	− 0.4 (− 1.18, 0.37)	0.304
Percentage VAS difference between pre-injection and post-injection 15 min	− 50.67 ± 26.43	− 43.06 ± 23.5	− 7.62 (− 20.45, 5.21)	0.24
VAS during activity at 2 weeks	2.59 ± 1.79	2.58 ± 1.18	0.01 (-0.75, 0.78)	0.973
VAS during activity at 1 month	2.47 ± 1.46	2.61 ± 1.43	− 0.14 (− 0.87, 0.58)	0.694
VAS during activity at 3 months	2.81 ± 1.26	3.06 ± 1.61	− 0.25 (− 0.98, 0.47)	0.49
VAS during activity at 6 months	3.56 ± 2.46	3.03 ± 1.62	0.53 (− 0.52, 1.58)	0.316
WORC during activity at 1 month	73.05 ± 17.93	72.73 ± 11.63	0.32 (− 7.32, 7.96)	0.934
WORC during activity at 3 months	73.59 ± 13.68	73.66 ± 11.89	− 0.07 (− 6.53, 6.4)	0.984
WORC during activity at 6 months	72.15 ± 18.5	70.3 ± 21.12	1.85 (− 8.14, 11.84)	0.712
DASH during activity at 1 months	26.02 ± 16.23	23.68 ± 9.88	2.34 (− 4.46, 9.13)	0.495
DASH during activity at 3 months	27.1 ± 15.52	27.94 ± 13.76	− 0.84 (− 8.24, 6.56)	0.821
DASH during activity at 6 months	24.4 ± 16.19	28.11 ± 19.29	− 3.71 (− 12.67, 5.25)	0.41

### Secondary outcomes

#### VAS

**Figure 4:**
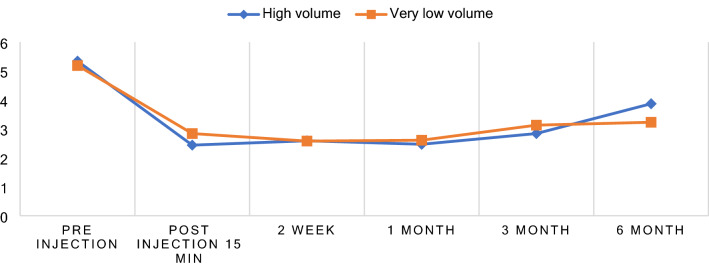
Change of the VAS referred by patient before and after the SCI.

15 min after SCI, 2 weeks and 1, 3- and 6-months follow-up, both the HVCI group and the VLVCI group showed a significant decrease in the VAS scores for pain with during activities. Using the Generalized Estimating Equation, we compared the two continuous outcomes, namely, pain VAS and WORC scores between groups (GEE). The statistical analysis in VAS, set with a significant difference level, was less than 0.05. The VAS score for pain during activities decreased from 4.91 ± 2.52 before the injection, to 2.61 ± 1.8, 15 min post injection (mean decrease of 2.3 (95% CI = − 3.33 to − 1.28) in the high-volume corticosteroid injection group (P < 0.001) and the very low volume corticosteroid injection group decreased from 4.78 ± 2.3 to 2.78 ± 1.59 (95% CI = − 2.75 to − 1.25), respectively, for both groups (P-value < 0.001). Fifteen minutes post-injection, there was a similar trend in the efficacy, in the lowering in the pain score between the treatment groups (P _mean difference VAS_ = 0.324, P _percentage VAS difference_ = 0.24) (Table 3). At 2 weeks, and the 1-, 3- and 6-months follow-up, the VAS score for pain during activities within both of groups was significantly decreased in comparison to the score before the injection in the HVCI group and the VLVCI group (P < 0.001) (Table 2). Both the HVCI and the VLVCI groups showed an equivalent improvement in VAS relative to the baseline values at all time points following SCI, with no significant difference in the mean decrease between the groups (Table 3, Fig. 4).

#### DASH

**Figure 5:**
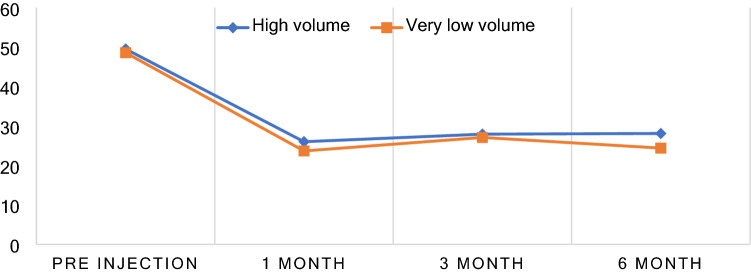
Mean DASH score by the progression time to treatment with SCI.

For the DASH during activity, there were no significant differences between groups at baseline. The data from DASH were analyzed using the Generalized Estimating Equation (GEE), and the P-value was found to be significantly differenced to set at less than 0.005. Both the HVCI group and VLVCI group had significant decreases in all scores at 1, 3 and 6 months, compared to the baseline (Table 2); however, there was no significant difference in the change from baseline between the groups (Table 3, Fig. 5).

#### ROM

There were no significant increases or decreases in range of motion (forward elevation and external rotation) in either of the groups at 2 weeks, and 1, 3, and 6 months compared with baseline (pre-injection), and no significant differences between the groups (Table 4). The variables with normal distribution were analyzed with the independent T-test, The analysis of the data was based on the follow-up outcome scores compared to those for pre-injection. The level of significance was determined as P < 0.05.

**Table 4:** Range of motion at pre-injection times, 2 weeks and 1-, 3- and 6-months follow-up.

		High volume (n = 32)	Very low volume (n = 31)	*P* value
Forward flexion	Pre-injection	154.38 ± 18.31	156.13 ± 18.01	0.703
	2 weeks	161.56 ± 15.26	164.19 ± 11.48	0.444
	1 month	163.13 ± 10.91	162.58 ± 9.65	0.835
	3 months	160.63 ± 11.62	159.68 ± 11.1	0.742
	6 months	153.44 ± 15.1	156.77 ± 13.01	0.352
External rotation	Pre-injection	71.72 ± 11.54	75.16 ± 13.81	0.287
	2 weeks	72.5 ± 14.48	76.45 ± 13.49	0.267
	1 month	72.66 ± 14.42	73.23 ± 12.15	0.866
	3 months	70.94 ± 12.85	68.87 ± 13.64	0.538
	6 months	59.38 ± 18.83	60.16 ± 17	0.863

Regarding complications 15 min post-injection, one patient from the HVCI group was subject to vasovagal syncope. However, this disappeared after 30 min, during observation at the orthopedics OPD. For those patients who received the subacromial corticosteroid injection, no association was apparent between the HVCI and the VLVCI group related to any complications i.e., Skin hypopigmentation or other Infection.

## Discussion

The VLVCI group's outcome was similar to that of the HVCI group's, indicating that it might be used routinely in treating subacromial impingement syndrome. We found that the VLVCI had the same therapeutic and diagnostic effect as those of the HVCI, which has been a commonly used standard in general orthopedics. It was found that for the VLVCT group, 15 min post-injection of HVCI of mixed lidocaine solution, lidocaine overdose in blood serum could cause allergy, vasovagal syncope, or severe consequence complications. The difference in the VAS percentage after 15 min was not significant, indicating that the VLVCI could be used as an impingement test in the same way that a traditional impingement test with a main lidocaine solution component would be used. There has never been a study comparing VLVCI or pure corticosteroid without a mixed anesthetic solution. This is the first study to use VLVCI to increase the most potential steroid concentration effect, while avoiding the complications associated with lidocaine, the main component of HVCI and other different volume SCI comparative studies.

Because the cause of SIS, both intrinsic and extrinsic, is anatomical structural changes that affect the subacromial space, the rehabilitation program is initiated as the first choice of treatment. Especially with secondary impingement, which is primarily caused by muscular imbalance, a rehabilitation program that includes exercise therapy (therapeutic exercise) and manual therapy will encourage patients to maintain and restore normal ROM of the shoulder girdle, which includes glenohumeral mobilization techniques, motor control exercises, scapular stabilization, and stretching^10,11^.

Triamcinolone acetonide (TMC) is a kind of corticosteroid that is commonly used in orthopedic procedures. Through reducing inflammatory mediators and influencing the cells engaged in inflammatory responses, the therapeutic effects affected both anti-inflammation and direct analgesic benefits^12^. This study assessed the efficacy of corticosteroid (TMC) injections at two different volumes, which are the most commonly utilized in subacromial injections for patients with subacromial impingement syndrome. Short-half-life steroids like TMC are often used in very low-volume corticosteroid injections. Despite the fact that the general results of the steroid shoulder injection in instances of shoulder pain are deemed good at this time. The injection of 1 ml (40 mg) TMC, both without lidocaine in addition to assessing mainly the response to the corticosteroid, resulted in improved range of motion (33 percent) and pain relief (61 percent) at the 2-week follow-up. From the theory, the onset of the action of corticosteroid is 24–48 h, and the duration of action is approximately 2–3 weeks^13^. The short-term pharmacologic action of TMC in therapy seems to have a more rapid effect response, as evidenced by a comparison study of methylprednisolone (MTP) and triamcinolone (TMC), MTP or TMC, both without lidocaine, in evaluating only the corticosteroid response. Corticosteroid injections were delivered into the subacromial space in this study. When compared to the VAS pre-injection record, the VAS improved by 43 percent at 10 min post-injection, and the ROM improved by about 25 percent in TMC groups^14^ even without the use of lidocaine, commonly combined with corticosteroids injections in pain relief in patients with shoulder pain due to be related to the TMC's high anti-inflammatory potency^15^.

The signs and symptoms of lidocaine intoxication, which rely on plasma levels larger than 5 mcg/ml, include slurred speech, tinnitus, circumoral paresthesia, and feeling faint, are difficult to predict. If the concentration is higher than 10 mcg/ml, the patient may experience seizures or lose consciousness. At 15 mcg/ml, the heart and central nervous system become more depressed, leading to cardiac arrhythmias, respiratory arrest, and cardiac arrest at 20 mcg/ml^16^. Furthermore, an aminoamide local anesthetic group impaired the rotator cuff tendon fibroblast's cytotoxic mechanism and caused cell death by increasing the generation of reactive oxygen species, such as ropivacaine, bupivacaine, and lidocaine^17,18^. Corticosteroid injections can also induce transient pain, skin atrophy, depigmentation, and septic arthritis, as well as have detrimental effects on intra-articular cartilage or tendon degeneration and even tendon ruptures^19^. When compared to low-volume CI, however, high-volume CI of lidocaine is associated with a higher risk of consequences (soft tissue irritation). Likewise, when corticosteroids are at lower concentrations, the steroid effect decreases. Subacromial injection was developed to reach a high drug concentration at the site of pathology while using less overall drug to avoid systemic side effects^6^. Currently, a combination of corticosteroid solution and local anesthetics is injected into a local soft tissue inflammatory site in clinical practice. However, the appropriate dosage, concentration, and volume in the SCI are still debatable. According to the study results of our previous systematic review and meta-analysis, high volumes (greater than or equal to 5 cc) of lidocaine in combination with corticosteroids for injection in subacromial impingement syndrome had a lower ASES, pain VAS, and risk of complications when compared to lower volumes (less than 5 cc). Low-volume CI may be a viable alternative as an injectable agent in subacromial impingement syndrome, considering its non-inferiority in terms of pain VAS score^9^.

Many subacromial injection plans for SIS patients, including those in this study for NSIAD, mentioned that a posterolateral blind injection approach compares 60 mg of Ketolac injection to 40 mg of triamcinolone in shoulder impingement syndrome. As the result of the ROM and functional score outcomes, the Ketolac group at 4 weeks is superior to triamcinolone in terms of UCLA shoulder rating score and active abduction (20 versus 40 respectively (P = 0.03), 134 versus 151 degrees respectively (P = 0.03)) because of the longer half-life of the drug. However, regarding effectiveness, both groups can significantly improve and achieve satisfying outcomes^20^.

The role of ultrasonography is widely used in the shoulder region, both extraarticular and intraarticular, for diagnosis, intervention, and biomechanical research^21^. According to this study, there was no effect on the properties of the rotator cuff and biceps tendon from a difference in corticosteroid dose (triamcinolone 40 mg versus 20 mg) administered by ultrasound-guided injection at the peritendinous and intrabursal sites. The first group receives standard subacromial-subdeltoid bursa injection; the second group uses dual target injection by injecting half the dosage at the supraspinatus and the other half at the bicep tendon; and the third group receives no injection. There is no evidence that the difference in corticosteroid dosage (40 mg vs. 20 mg) will affect the elasticity of the supraspinatus and bicep tendon by strain ratio ultrasound evaluation because the injections have a minimal effect on the mechanical properties of the tendon or because the corticosteroid injections have transient and reversible effects^22^. Dynamic ultrasound is used to improve the biomechanical estimation of the vertical acromiohumeral distance (AHD), rotation radius, and humeral head rotation angle. The study's goal purposed to observe if there was a minimal decrease in vertical AHD in cases of subacromial impingement syndrome, which would lead to rotator cuff pathology in the future due to the decreased minimal vertical AHD during the dynamic ultrasound examination while there were abnormal changes in subacromial motion metrics^23^.

For subacromial impingement syndrome, the clinical outcome of ultrasound-guided subacromial injections was compared to blind subacromial injections. After a short-term follow-up, blind injections into the subacromial bursa were just as successful as ultrasound-guided injections in reducing pain and function in subacromial impingement syndrome. As a result, ultrasonography guiding for subacromial injections is no longer required^24^. The difference in injection precision, on the other hand, could be a source of heterogeneity. There is no significant difference in pain between high-corticosteroid injections using ultrasound guidance or landmark-guided injections using the posterior approach injection technique^25^. When comparing low-corticosteroid injections with ultrasound guidance to low-corticosteroid injections with landmark guidance, there is a significant difference in pain. As a result, we recommend using an ultrasound-guided approach for low corticosteroid injections to improve the outcome of corticosteroid injections in subacromial impingement syndrome, or a landmark-guided technique can be used in the case of high corticosteroid injections^6^.

Our study's strengths include a prospective randomized control trial, long-term and short-term follow-up, and the use of a standardized and validated VAS, WORC, and DASH scores. This is the first study to compare the efficacy of corticosteroid injections in the subacromial impingement syndrome using HVCI and VLVCI (pure corticosteroid injection without analgesic solution combination). Data regarding all possible adverse effects were also collected. The follow-up rate was of a reasonably standard range, at approximately 85 percent in both of groups. We only had one patient that we were unable to include, due to the patient's decision to undergo the procedure on her own. Across all samples, less than 5 percent of participants to dropped out. In treatment of subacromial impingement syndrome. The follow-up period was short to long-term (6 months). Hence, the long-term effectiveness and adverse effects of treatment could be assessed.

The study's limitations were that firstly, the blinding technique was ineffective, as only assessors were used, and secondly, the trial was single-blinded, which may have resulted in underpowering for the primary and secondary outcomes. Different surgeons administered the corticosteroid injections. Although some publications suggested that low-volume corticosteroid injection (less than 5 ml) be utilized with the USG technique in low-CI, in order to improve the outcome, we only had a limited medical device and landmark guide injection was still a widespread practice among orthopedists in routine practice. Moreover, the lack of ultrasound guidance so the precise positioning of the injection is unclear.

## Conclusions

This study comparing VLVCI and HVCI found that VLVCI are non-inferior to HVCI in the primary and secondary endpoints of pain VAS score during activity. Furthermore, there was found to be no statistically significant difference in the clinical shoulder function ratings (WORC and DASH score) following subacromial injection with VLVCI versus HVCI. However, there were still cases of vasovagal syncope in the high-volume corticosteroid injection group, but no soft tissue irritation (infection, skin discoloration, or allergic reaction) in either both groups.

## Data Availability

The authors confirm that the data supporting the findings of this study are available within the article and its supplementary material.
